# A comparative study of 21,194 UKAs and 49,270 HTOs for the risk of unanticipated events in mid-age patients from the national claims data in South Korea

**DOI:** 10.1186/s12891-022-05080-8

**Published:** 2022-02-08

**Authors:** Sun-Ho Lee, Hae-Rim Kim, Hyoung-Yeon Seo, Jong-Keun Seon

**Affiliations:** 1 Department of Orthopedic Surgery, Chonnam National University Medical School and Hospital, 322 Seoyang-ro, Hwasun-eup, Hwasun-gun, Jeollanam-do Republic of Korea; 2 College of Natural Science, School of Statistics, University of Seoul, Seoul, Republic of Korea

**Keywords:** Knee, Osteoarthritis, High tibial osteotomy, Arthroplasty, Revision, Nationwide

## Abstract

**Background:**

Both high tibial osteotomy (HTO) and unicompartmental knee arthroplasty (UKA) are well-established treatments for medial knee osteoarthritis (OA). However, over the past 20 years, results of comparisons of long-term survival rates and outcomes have remained controversial. Furthermore, in patients at the boundary age, from 50 to 70 years, considering age as a treatment indication, selecting a surgical method is difficult. Therefore, we aimed to investigate conversion rates to total knee arthroplasty (TKA) and perioperative adverse outcomes between the two surgical methods in mid-age patients.

**Methods:**

We extracted data from the Korean National Health Insurance claims database. A total of 70,464 patients aged between 50 and 70 years, considered as mid-age patients were included in the final study population. We used a multivariable Cox proportional hazard regression model, adjusting for potential confounders such as age, sex, insurance type, region of residence, hospital type, comorbidities, and the Charlson comorbidity Index (CCI).

**Results:**

Of the 70,464 patients, 21,194 were treated with UKA and 49,270 were treated with HTO. HTO showed a higher risk of revision than UKA at five, and 10 years and during the whole observation period. The incidence of deep vein thromboembolism, and surgical site infection was significantly higher in UKA than in HTO.

**Conclusions:**

It is important to choose an appropriate surgical method considering that UKA has better results in terms of long-term survival rates but may have a higher incidence of various complications.

## Background

Most patients with knee osteoarthritis(OA) have lesions limited to the medial compartment, and high tibial osteotomy (HTO) and unicompartmental knee arthroplasty (UKA) are well-established treatments for this condition [1–3]. HTO and UKA, which are often performed as primary surgeries in medial knee OA, report good results as the ultimate treatment for OA with an added advantage of delaying conversion to total knee arthroplasty (TKA) [4, 5]. However, to date, considering which of the two surgical methods to choose remains controversial, comparing long-term survival rates and surgical outcomes [6–8] In particular, when selecting a surgical method for patients in their 50 s and 60 s, there are many considerations. [3, 9–11]. Both HTO and UKA have the advantage of saving native joint spaces, but there are some differences in surgical techniques and indications. HTO is considered primarily in young and active patients, and has the advantage of preserving the native knee compartment by correcting malalignment of the lower extremity and shifting the axis of the weight load to the lateral compartment [6, 12]. Good results have been reported for UKA in relatively elderly patients with less physical requirements, by replacing only the medial compartment in which the arthritic change was progressing [13]. Due to factors such as improvement of surgical methods and implants, surgical indications between the two operations have overlapped [6, 14]. In addition to age and physical demand, the relative merits of the two surgical methods are unclear and are still controversial which lead a lack of clear criteria for selecting a surgical method [6]. Numerous studies have compared the advantages and clinical results between the surgical methods, and the superiority of one over the other has not been proven [1–8, 12–14]. Therefore, the objectives of this study are as follows: (1) to evaluate revision rates of the two surgical methods and (2) analyze the outcomes in terms of perioperative complications. It was hypothesized that there is no difference between HTO and UKA with regard to survival and complication rates because both treatments are well established treatment methods.

## Methods

### Data sources

This study was conducted using data from the Korea National Health Insurance Review and Assessment Service. Korea's health insurance system is considered well-established worldwide, with almost 99% of the population enrolled [15]. The Korean health insurance claim data uses the International Statistical Classification of Diseases and Related Health Problems, Tenth Revision (ICD-10) codes and Electronic Data Interchange (EDI) codes, which are internationally accepted classifications for diagnosis and procedures. Information such as patient's age, gender, diagnosis, hospitalization record, surgery record, drug prescription, and hospital area information is provided in an anonymous form, and the individual data linked to each code are recorded in the NHI database in Korea. Data were obtained from January 2008 to May 31 2019. All medical records have been provided for patients diagnosed with osteoarthritis of the knee joint and having undergone surgical treatments.

### Study approval

This study was approved by the Institutional Review Board of Chonnam National University Hwasun Hospital. The review board waived the requirement for patient consent considering the characteristics of this study.

### Data collection

**Fig. 1:**
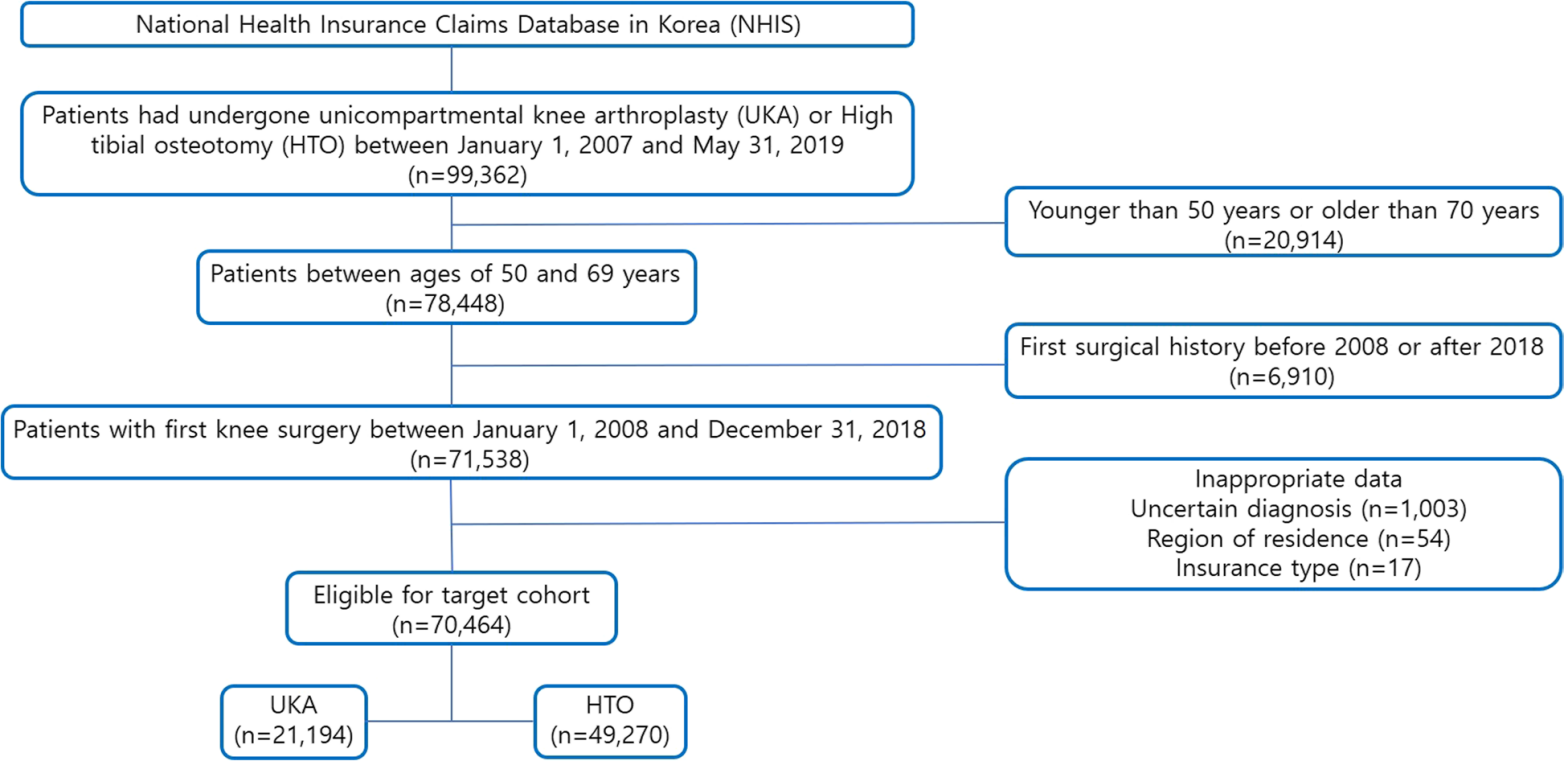
Flowchart for target population

The diagnostic code for knee OA (M17) and procedure codes for UKA (N2712, N2717) or HTO (N0304) were used to extract patients who had undergone UKA or HTO for the treatment of knee OA. Patients under 50 years of age and over 70 years of age were excluded for analysis of middle-aged participants. An effort was made to extract newly diagnosed and operated patients within a defined study period by applying a 1-year wash-out period for knee arthritis and surgical treatment history (Fig. 1). We compared UKA and HTO in terms of conversion to primary TKA, incidence of unanticipated major medical problems, Intensive Care Unit (ICU) admission, postoperative transfusion, and readmission rates. Patient demographic data were extracted using the aforementioned diagnostic code and each surgical code and included age, sex, type of insurance, hospital, region of residence, and medical comorbidities (Table 1). By using the Korean claim data, it was not possible to distinguish between medial open wedge osteotomy and lateral closed wedge osteotomy of HTO and the type of implant. The type of hospital was classified as a teaching hospital(> 500 beds), a general hospital(30–500 beds), an independent hospital(< 30beds), and a private clinic(outpatient clinic). The region of residence was classified, based on population, as cities with a population of 10 million or more(e.g., Seoul), cities with a population of 1 million or more(eg, Busan, Incheon, Daegu, Gwangju, Daejeon and Ulsan), and cities with a population of less than 1 million(Gyeonggi, Gangwon, Gyeongsangbuk, Gyeongsangnam, Chungcheongbuk, Chungcheongnam, Jeollabuk, Jeollanam and Jeju). Medical comorbidities (hypertension, diabetes mellitus, hyperlipidemia, osteoporosis, peripheral vascular disease, depression and dementia) were confirmed based on the ICD-10 diagnostic codes with at least two claims within 1 year from the date of surgery. The Charlson comorbidity index (CCI) score was calculated from the ICD-10 codes by introducing previous literature methods. Statistical analysis was performed to consider as many adjusted variables as possible to address the imbalance of basic patient characteristics between the two groups.

**Table 1:** Patient baseline characteristics in 50–69 aged patients

	UKA	HTO	SMD
	(*N* = 21,194)	(*N* = 49,270)	
Age (mean (sd))	60.41 (5.09)	57.84 (4.73)	0.523
Sex (%)			0.178
Female	17,365 (81.9)	37,845 (76.8)	
Male	3829 (18.1)	11,425 (23.2)	
Hypertension (%)	12,066 (56.9)	23,313 (47.3)	0.193
Hyperlipidemia (%)	12,101 (57.1)	26,843 (54.5)	0.053
Peripheral vascular disease(%)	6011 (28.4)	11,813 (24.0)	0.1
Diabetes_with out complication (%)	6352 (30.0)	12,246 (24.9)	0.115
Diabetes_with complication (%)	2582 (12.2)	4600 ( 9.3)	0.092
Osteoporosis (%)	7890 (37.2)	13,907 (28.2)	0.193
Depression (%)	3819 (18.0)	8335 (16.9)	0.029
Dementia (%)	392 ( 1.8)	611 ( 1.2)	0.049
Type of insurance (%)			0.004
Health insurance	20,504 (96.7)	47,697 (96.8)	
Medical benefits	690 ( 3.3)	1573 ( 3.2)	
City of residence (%)			0.163
Over 10milion	7452 (35.2)	13,967 (28.3)	
Over 1milion	4787 (22.6)	13,780 (28.0)	
Others	8955 (42.3)	21,523 (43.7)	
Type of hospital (%)			0.122
Teaching hospital	2016 ( 9.5)	4940 (10.0)	
General hospital	4625 (21.8)	8437 (17.1)	
Independent hospital	13,809 (65.2)	33,774 (68.5)	
Private clinic	744 ( 3.5)	2119 ( 4.3)	
CCI (%)			0.11
0	1301 ( 6.1	3789 ( 7.7)	
1	3072 (14.5)	8152 (16.5)	
2	4284 (20.2)	10,731 (21.8)	
≥ 3	12,537 (59.2)	26,598 (54.0)	

*UKA* Unicompartmental knee arthroplasty, *HTO* High tibial osteotomy, *SMD* Standardised mean difference, *CCI* Charlson comorbidity index

^*^CCI: Myocardial infarction, Congestive heart failure, Peripheral vascular disease, Cerebrovascular disease, Dementia, Chronic pulmonary disease, Connective tissue disease, Peptic ulcer disease, Mild liver disease, Moderate or severe liver disease (3), Diabetes without complications, Diabetes with complications (2), Paraplegia and hemiplegia (2), Renal disease (2), Cancer (2), Metastatic carcinoma (6), AIDS/HIV (6)

### Statistical analysis

The baseline characteristics of patients treated with HTO or UKA were summarized using descriptive statistics, including proportion, mean, and standard deviation. Differences between UKA and HTO in continuous variables were evaluated with analysis of variance and categorical variables were compared using the Chi-square test. Wilcoxon statistic assigned a greater weight to differences occurring near the beginning of the study. The imbalance in baseline characteristics between the two groups was evaluated with standardized mean difference (SMD). An SMD of less than 0.1 was considered a negligible difference between the groups. Covariants with an SMD greater than 0.1 were corrected by setting them as adjusting variables. The person-years (PY) for each group of patients were calculated from the date of primary surgery to the event of subsequent revision and various adverse outcomes. We conducted a stratified log-rank test and obtained Kaplan–Meier curves considering potential confounders. In addition, conditional logistic and stratified Cox regression analyses were conducted to calculate complication rates. The adjusted hazard ratio (HR) and 95% confidence interval (CI) were calculated using a multivariable Cox proportional hazard regression model adjusting for potential confounders such as age, sex, insurance type, region of residence, hospital type, comorbidities, and the CCI. All statistical analyses were performed using R software (version 3.4.1; R Foundation for Statistical Computing) and SAS Enterprise software (version 6.1; SAS Institute).

## Results

According to the data extracted through Korea's Health Insurance Review and Assessment Service from January 2007 to May 31, 2019, a total of 78,448 patients aged 50 to 70 who underwent UKA or HTO were recorded. Considering the one-year wash-out period, 6,910 patients with surgical records before 2008 and after 2018 were excluded, and 1,074 patients with inappropriate claims data were eliminated, therefore, 70,464 patients were included as the final study subjects, of which 21,194 were in the UKA group and 49,270 in the HTO group (Fig. 1). The mean age of the patients was 60.4 years in the UKA group and 57.8 years in the HTO group, meanwhile, the proportion of women in both groups was remarkably high. Both treatments showed an increase in the number of surgeries over the years, and in terms of underlying disease characteristics, hypertension, diabetes, and osteoporosis were more common in the UKA group, which had a higher average age (Table 1).

**Fig. 2:**
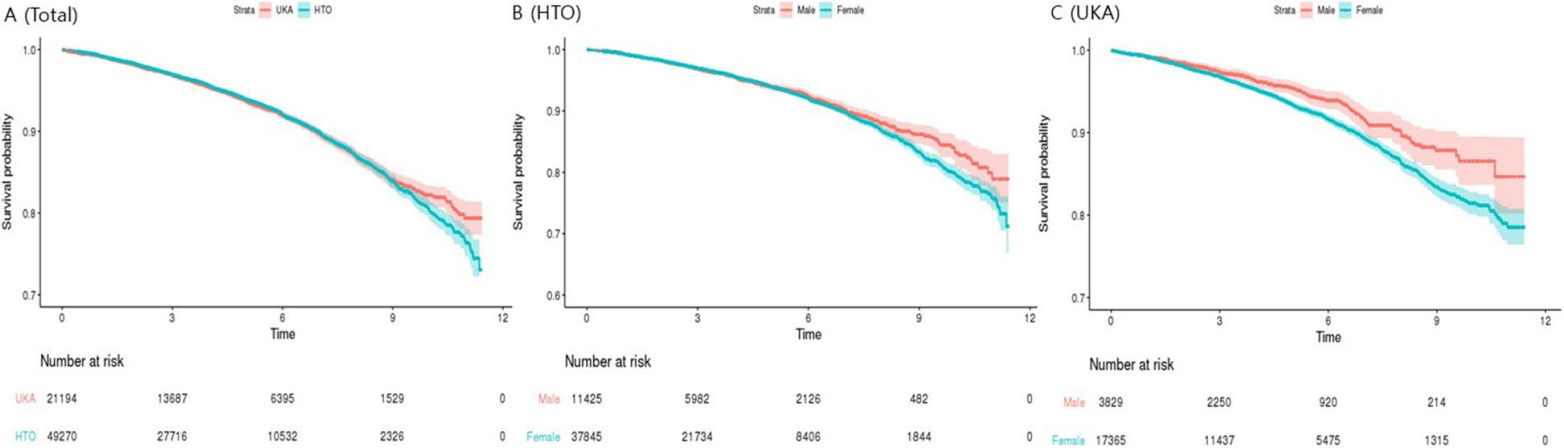
Kaplan–Meier survivorship curve: 11.5-year survival probability of total patients (**A**), gender differences in HTO (**B**), gender differences in UKA (**C**)

There were no significant differences in an unadjusted analysis when comparing the risk of requiring revision between the two groups (Table 2). The Kaplan–Meier survivorship curve showed no significant difference between UKA and HTO (*p* = 0.92) (Fig. 2A), and in the survival rate in the HTO group according to sex (*p* = 0.16) (Fig. 2B), but in the UKA group, the survival rate was higher in male patients than in female patients (*p* < 0.001) (Fig. 2C).

**Table 2:** COX proportional hazard survival analysis for risk of revision for 50–69 aged patients

		HTO(*n* = 49,270)			UKA (*n* = 21,194)			Crude			adjusted*		
		N	%	1,000 PY	N	%	1,000 PY	HR	95% CI	*P*-value	HR	95% CI	*P*-value
Revision	event	2674	(5.43%)	13.88	1402	(6.62%)	14.70	1.00	(0.94, 1.07)	0.92	1.19	(1.11,1.27)	< .0001
	days	1433.39	± 953.23		1474.69	± 930.66							
Revision(5 year)	event	1819	(3.69%)	11.34	913	(4.31%)	12.10	0.96	(0.89, 1.04)	0.31	1.17	(1.07,1.27)	0.0003
	days	884.88	± 500.84		905.48	± 520.35							
Revision(10 year)	event	2630	(5.34%)	13.71	1390	(6.56%)	14.65	1.00	(0.93, 1.06)	0.88	1.18	(1.10,1.26)	< .0001
	days	1392.99	± 907.90		1454.19	± 907.97							

^*^adjusted variable: age, sex, comobidities, type of insurance, type of hospital, region of residence, CCI

*UKA* Unicompartmental knee arthroplasty, *HTO* High tibial osteotomy, *HR* Hazard ratio, *CI* Confidence Interval, *PY*: person year, reference: UKA

An adjusted analysis considering baseline characteristics such as age, sex, comorbidities, insurance type, hospital size, residence, and the CCI, showed that the risk of requiring revision in HTO was higher than that in UKA (HR: 1.19, 95% CI: 1.11–1.27) (Table 2). The HRs were 1.17 (1.07–1.27) at 5 years and 1.18 (1.10–1.26) at 10 years. A Cox proportional hazard analysis revealed that the incidence of deep vein thromboembolism (DVT) (HR: 0.33, 95% CI: 0.28–0.39), and surgical site infection (HR: 0.40, 95% CI: 0.35–0.46) was significantly higher in UKA than in HTO (Table 3). Other adverse outcomes, including pulmonary thromboembolism, cerebrovascular disease, myocardial infarction, postoperative delirium, and acute renal failure (ARF), showed no significant differences. In terms of perioperative complications, the incidence of postoperative ICU admission was significantly higher in UKA (OR: 0.21, 95% CI: 0.15–0.29), while that of re-hospitalization within 30 days (OR: 1.28, 95% CI: 1.18–1.38) and 90 days (OR: 1.28, 95% CI: 1.22–1.34) was higher in HTO (Table 4).

**Table 3:** COX proportional hazard analysis for adverse outcomes

		**HTO(*n* = 49,270)**			**UKA (*n* = 21,194)**			**Crude**			adjusted*		
		**N**	**%**	**1,000 PY**	**N**	**%**	**1,000 PY**	**HR**	**95% CI**	***P*-value**	**HR**	**95% CI**	***P*-value**
Deep vein thromboembolism	events	251	(0.51%)	1.30	394	(1.86%)	4.13	0.29	(0.25, 0.34)	< .0001	0.33	(0.28,0.39)	< .0001
	days	738.35	± 923.41		303.32	± 659.29							
Pulmonary thromboembolism	events	113	(0.23%)	0.59	87	(0.41%)	0.91	0.62	(0.47, 0.82)	0.001	0.75	(0.56,1.01)	0.06
	days	557.09	± 775.84		850.03	± 1045.18							
Cerebrovascular disease	events	6475	(13.14%)	33.61	3699	(17.45%)	38.79	0.83	(0.80, 0.87)	< .0001	1.02	(0.98,1.06)	0.36
	days	802.38	± 828.81		874.19	± 867.41							
Myocardial infarction	events	391	(0.79%)	2.03	231	(1.09%)	2.42	0.83	(0.71, 0.98)	0.03	0.88	(0.74,1.04)	0.12
	days	880.55	± 916.71		1024.94	± 1000.18							
Acute renal failure	events	238	(0.48%)	1.24	167	(0.79%)	1.75	0.72	(0.59, 0.88)	0.001	0.81	(0.66,1.02)	0.08
	Days	1042.95	± 964.47		1225.66	± 1029.08							
Postoperative Delirium	events	40	(0.08%)	0.21	32	(0.15%)	0.34	0.65	(0.41, 1.04)	0.07	0.97	(0.59,1.59)	0.90
	Days	1169.35	± 924.57		1479.34	± 1072.69							
Surgical site infection	events	400	(0.81%)	2.08	483	(2.28%)	5.06	0.38	(0.33, 0.44)	< .0001	0.40	(0.35, 0.46)	< .0001
	days	541.42	± 777.47		558.08	± 785.58							

^*^adjusted variable: age, sex, comobidities, type of insurance, type of hospital, region of residence, CCI

*UKA* Unicompartmental knee arthroplasty, *HTO* High tibial osteotomy, *HR* Hazard ratio, *CI* Confidence Interval, *PY* person year, reference: UKA

**Table 4:** Logistic regression analysis for perioperative complications

	**HTO(*n* = 49,270)**		**UKA (*n* = 21,194)**		**Univariate analysis**			**Multivariable analysis (adjusted)**		
	**n**	**(%)**	**n**	**(%)**	**OR**	**95% CI**	***P*-value**	**OR**	**95% CI**	***P*-value**
ICU	48	(0.10%)	109	(0.51%)	0.19	(0.13, 0.27)	< .0001	0.21	(0.15,0.29)	< .0001
Blood transfusion	2	(0.00%)	4	(0.02%)	0.22	(0.04, 1.17)	0.08	0.24	(0.04,1.39)	0.11
Rehospital(30 days)	2601	(5.28%)	853	(4.02%)	1.33	(1.23, 1.44)	< .0001	1.28	(1.18,1.38)	< .0001
Rehospital(90 days)	7978	(16.19%)	2692	(12.70%)	1.33	(1.27, 1.39)	< .0001	1.28	(1.22,1.34)	< .0001

Odd ratios less than 1 favour high tibial osteotomy

*UKA* Unicompartmental knee arthroplasty, *HTO* High tibial osteotomy, *ICU* Intensive Care Unit, *OR* Odd Ratio, *CI* Confidence Interval

## Discussion

We identified 78,448 patients who underwent UKA or HTO, to evaluate the results regarding the revision rate and postoperative complications relevant to each treatment. Throughout the total observation period of 11.5 years, the survival rate after UKA was significantly higher than that after HTO. In addition, when comparing the risk of complications after surgery, some significant differences were found between the two surgeries. Notably, the incidences of postoperative DVT and surgical site infection were higher in UKA than in HTO.

In previous studies on the survival rates of HTO and UKA, different results have been reported over the past 20 years. Some reported that UKA had better long-term survival rates compared to HTO [8, 16]. On the contrary, other studies showed better results for HTO [17], meanwhile, other studies concluded that there were no significant differences [5, 6, 13]. However, most of the previous comparison studies consisted of different patient characteristics, and short retrospective or small randomized controlled studies. In the present study, the long-term survival rate was analyzed, with adequate power and using adjusted covariates, and the risk of revision was compared at different time points, that is at 5 and 10 years.

In terms of postoperative complications, various results are reported in the literature. Some researchers reported that UKA is superior to HTO in terms of postoperative function and has fewer complications [6, 13, 18, 19], while others reported little differences between the two treatments [4, 14, 20]. However, to the best of our knowledge, the incidence of the major postoperative medical complications such as pulmonary, cerebrovascular and cardiac problems has not been compared between HTO and UKA, unlike a study exists in TKA and UKA [21]. We found no significant difference between the two surgeries in most complication rates, meanwhile, significant differences were found in the incidence of DVT and surgical site infection. The HTO group showed significantly low infection rate, which may be related to a relatively short operation time [22–24] and showed low risk of DVT possibly due to routine usage of mechanical compression after surgery [25], and pharmacologic prophylaxis [26]. There have been previous studies on factors related to ARF after orthopedic surgery, but studies related to different surgical methods, especially artificial joint surgery, have been insufficient and may require further evaluation [27, 28]. On the other hand, cemented knee arthroplasty, as one of the independent risk factors for postoperative ICU admission in some studies could relate that the result of higher ICU admission in UKA as shown in Table 4 [29, 30]. HTO often requires a more dependent functional status postoperatively compared to UKA, which may be a reason for higher re-hospitalization in HTO [2, 7, 31, 32].

In this study, the survival rate and complications of UKA and HTO were analyzed for mid-aged patients, but as the indications of UKA and HTO widened and overlapped [33–35], there are many reports of good results obtained by performing UKA and HTO in a younger patient group [10, 11]. However, there are still studies showing that the risk of early conversion to TKA increases as the patient's age increases [9, 34, 36]. In contrast, Other studies concluded that the relationship between age and implant survival and clinical outcome is not significant [35, 37–39]. they suggest that UKA and HTO should be performed regardless of age. Elderly or young age should not be a contraindication for selecting surgical methods [35, 37–39].

The strength of our study is the use of Korea's health insurance system which covers up to 99% of the entire population [15]. Using strict statistical analysis to reduce potential confounders, we carried out a long-term, large-scale population-based comparison study. However, this study had several limitations. First, the national registry data have inherent problems, including inaccurate diagnostic codes and lack of detailed medical records (e.g., type of inserted implants, different surgical techniques, and causes for revision surgery). Second, we could not identify the consecutive values such as body mass index, degree of leg deformity, and osteoarthritis stage. Furthermore, other clinical outcomes, including functional and radiological indicators were not provided. Third, the claim code of the health insurance claim data could not distinguish between the left and right side of the knee joint on which the procedure was done. Thus, patients who underwent the first knee replacement were defined when selecting subjects to analyze the comparative effects. The analysis included only those patients for whom there was only one claim for 'HTO or UKA' in the claim data, and the analysis excluded patients with two or more claims at different times because the exposure time could not be defined. Fourth, the maximum follow-up period in this study was 11.5 years which could be extended in future studies. In addition, there may be differences due to race and national differences compared to other countries. Despite these limitations, the authors believe that the current study is worthy because it is the first large-scale, long-term cohort study with patients of a specific age category, which is the boundary between the indications for the two surgeries.

## Conclusions

It is important to choose an appropriate surgical method for unicompartmental knee OA considering that UKA has better results in terms of long-term survival rates, but may have a higher incidence of various complications even considering the high prevalence of underlying diseases in the UKA group in the preoperative patient characteristics.

## Data Availability

The public access to the database is closed. Access to the database is granted only to researchers approved by the Korea Health Insurance Review and Assessment Service. All data is anonymized and encrypted to protect personal information, but external disclosure is prohibited in principle. Dataset: de-identified datasets generated and analysed during the present study will be made available by request from the Health Insurance & Assessment Service of Korea at https://opendata.hira.or.kr/. After user approaval by the Health Insurance Review and Assessment Service, a remote analysis system (https://ras.hira.or.kr) can be used by receiving a virtualized ID.

## Abbreviations

TKA: Total knee arthroplasty
UKA: Unicompartmental knee arthroplasty
HTO: High tibial osteotomy
OA: Osteoarthritis
CCI: Charlson comorbidity index
ICD: International statistical classification of diseases and related Health Problems
EDI: Electronic data interchange
ICU: Intensive care unit
SMD: Standardized mean difference
PSM: Propensity score matching
PY: Person-years
HR: Hazard ratio
CI: Confidence interval
DVT: Deep vein thromboembolism
